# Typhus Fever in Philadelphia in 1836

**Published:** 1837

**Authors:** W. W. Gerhard


### Art. II.—Typhus Fever in Philadelphia in 1836. By W. W. Gerhard, M. D.

In the American Journal for February, and August last, we have an account of an epidemic petechial Typhus fever, occurring the year before in Philadelphia, and chiefly at the Blockley Hospital, of which Dr. Gerhard is one of the physicians. We shall make such extracts from the second paper as may be useful to our practical readers.

"The glands of Peyer were found not merely free from the peculiar lesion occurring in dothinenteritis or typhoid fever, but these follicles and the rest of the intestine were more healthy in the petechial fever than in the majority of other diseases. We are the more certain of the state of these glands because our attention was closely directed to this subject, and we had previously made most numerous examinations of the glands in typhoid fever and in other diseases; we could therefore pronounce with certainty as to their actual condition. The mesenteric glands were always either normal or very little injected; and the spleen was altered only in one third of the whole number of patients.

The lesions of other organs were as various as they are in most

*To the kindness of Dr. William Thompson I have been chiefly indebted for the opportunities of consulting the different Theses I have had occasion to quote in the foregoing communication.

acute diseases, and evidently depended in a great degree upon the season, or upon the accidental circumstances in which the patient was placed. Thus, we had several cases of pneumonia in the months of March and April, while the lesions of the intestine were remarkably insignificant. But in the warmer season of June, July and August, diarrhœa was frequent, and was accompanied not by a lesion of the glands of Peyer, but by softening or other affections of the mucous coat of the colon.

The pathological anatomy of a disease will usually afford us useful data which may point out the analogies which unite it with other maladies. Now the absence of a permanent characteristic lesion, at least in the solids of the body, is one of the most remarkable peculiarities of the exanthemata. In all these diseases there is no constant lesion, with the exception of the eruption itself. The analogy is not limited to the pathological anatomy, as the subsequent part of this memoir will prove; and without wishing to lay too much stress upon the resemblances which may exist in some of the symptoms of a disease which presents a strong analogy, we cannot pass them by without due attention. As far, therefore, as the pathological anatomy will direct us, we are constrained to class typhus fever amongst the exanthemata."

As Dr. G. lays much stress on the efflorescence accompanying most cases of this fever, and as it seems, we had almost said, to have been but a new modification of scarlatina, we shall transcribe his account of it.

"An eruption of a peculiar character appeared on the skin of 32 out of 36 whites, in whom it was noted. Of the four cases in which it was not visible, one died upon the seventh day of the disease, and the others presented slight symptoms of fever, which disappeared in the course of four or five days. It was also visible, though less distinctly, in mulattoes; and we may infer that the color of the skin alone prevented its development in the negroes. It consisted of petechiæ, which in not more than six cases resembled the rose-colored spots of dothinenteritis. The petechiæ assumed the form of small reddish or purple spots from the breadth of one line, or even the eighth of an inch, down to that of a minute point. They were not elevated above the surface of the skin, and were without the regular round or oval shape of the rose-colored spots. The color of the petechiæ at first was of a lighter red, and could with difficulty be distinguished from the spots of typhoid fever; but on the second or third day they assumed the dull red or purple color of the eruption proper to the typhus; sometimes they were nearly black.

The eruption of petechiæ must therefore be regarded as a characteristic symptom, for it was present in nearly every case in which the symptom was sought for. The eruption is indeed quite as constant as that of measles or small pox; for those cases in which it could not be detected either died before the day on which it usually appeared, or were ephemeral, abortive cases of the disease, which ceased before passing through its regular periods. The petechiæ appeared from the sixth to the eighth day, after the beginning of the symptoms, and disappeared from the fourteenth to the twentieth day. But the time of their disappearance varied much, and the gradual diminution of their color rendered it difficult for us to indicate its precise date. The eruption would occasionally fade without entirely disappearing, and again resume its former color. This sudden and unexpected fading of the eruption coincided with a depression of strength, and was often of fatal prognosis. After death, a slight bluish ecchymosis, easily traced in the thickness of the true skin, indicated the place of the larger petechiæ, while the smaller were no longer visible. The regular progress of the eruption proves the close analogy which exists between typhus and some of the eruptive diseases, especially scarlatina and measles."

The following case will give an idea of the symptomatology and treatment of a mild case of this malady.

"Dr. F., one of the house physicians at the hospital, had been extremely devoted to his duties, and had spent much time in the ward in which many of the fever patients had been placed. At the time of his attack, the patients were in a great measure concentrated in this single ward. Dr. F. had resided in the house but a short time, and was of a full robust habit of body and in excellent health.

About the first of July, 1836, he found himself growing gradually weaker and appetite declining; but although he was sensible of a general feeling of uneasiness, he had no local pain except a dull, deep-seated aching in the centre of the forehead. There was no disturbance of any of the digestive functions. On the 7th he felt better, and took much exercise, in the hope of escaping any serious illness.

On the 8th, he went through his usual duties in the morning, but while in the ward, he was seized with a chill and nausea. These symptoms he referred to a furuncle situated on theright hand, between the finger and thumb. On the 9th, complained of cephalalgia, uneasiness at the stomach, and fever; he applied a poultice to his thumb, and took ten grains of calomel and one grain of ipecacuanha. Nausea and vomiting came on in an hour, and after drinking freely of warm water, he threw up a quantity of greenish bile. The next day he took a Seidlitz powder, which was followed by five evacuations. In the evening he attempted to resume his duties, but, from feebleness, discontinued them. From the ninth to the fourteenth the fever diminished every morning, but increased towards evening, so as to induce the patient to believe that he was attacked with ordinary remittent. He took the effervescing draught, and lived on light farinaceous diet.

14th. Restless during the night; almost no sleep: vomiting in the morning, but without pain. At 2 P. M., the pulse was full and rather forcible; skin hot and dry; tongue slightly furred; eyes injected; agitation and expression of general malaise. Twenty leeches were applied to the abdomen, followed by fomentations; the punctures bled until the next morning. Neutral mixture ℥ss. q. section h. cum ant. tart. gr. 1-24. In the evening felt better, but was very weak; no pain. Pulse 96, compressible, but of moderate volume. Skin hot and dry. Bowels open frequently during the day; had six watery stools, without pain. The diarrhœa ceased after an injection containing thirty drops of laudanum. A few rose-colored spots, slightly elevated and disappearing on pressure, were scattered over the abdomen.

15th. Slept well; no cephalalgia, but dizziness on rising. Eyes suffused; no tinnitus or deafness, countenance anxious. Anorexia; one dejection. Tongue clammy, pale, coated with a whitish fur; thirst, dryness of the fauces and fœtor of the breath; a similar odor, but less marked, exhales from the skin; short, dry cough. Pulse 98; skin hot and dry; numerous spots of a light red color, some slightly elevated and others on a level with the skin, disappear on pressure, and are scattered over the abdomen and extremities. Spt. mindereri. ℥ss. q. sec. h. Effervescing draught. Sponge surface with diluted solution of chloride of soda. Chloride of lime around the bed.

16th. Sleep disturbed by frightful dreams; same condition of the senses; intelligence and memory enfeebled. Eyes still suffused; purple flush of the face; less anorexia; tongue and thirst as yesterday. Abdomen a little tympanitic. Skin hot, but scarcely pungent. Petechiæ more numerous and of a more decided purple tinge than yesterday. Pulse 100, as yesterday. No stool until an enema containing a little chloride of lime was administered, when some consistent fæces were discharged. Urine reddish, cloudy. Same treatment; gruel.

17th. The petechiæ were larger and more livid on the arms. No sudamina or rose-colored spots. Extreme prostration; anxious, dull expression of countenance; dizziness of sight; sensibility of skin natural; delirium and insomnia. Pulse 112. Sponge with the solution every hour. Poultice, with solution of chloride of soda, to the abdomen; other treatment as before. During the next night he slept none, was very restless and delirious, and complained of much cephalalgia; cold applications were made to the head, sinapisms applied to the ankles, and gr. 1-8 of sulph. morph. given by one of his friends.

On the 18th the injection of the eyes and dulness of intellect had slightly increased, but he had slept for some hours after the morphia. Same treatment, adding two cold enemata.

19th. Petechiæ fading; skin less hot. Pulse 120, moderate volume. Memory more correct; abdominal functions natural; burning sensation at the stomach, after taking the acetate of ammonia. Substitute effervescing draught, and cold enema every three hours.

On the 20th, diarrhœa checked by an opiate enema. Mineral water instead of previous medicine. The skin had become more moist. Pulse less frequent.

On the 20th the pulse had fallen to 84 in the minute; the petechiæ finally disappeared, and the countenance assumed that haggard, wan appearance which succeeds the fever. The patient asks for food, which was now granted, with a little wine, and a few grains of the sulphate of quinine daily. The fever did not again return, although there was a slight diarrhœa; and after the convalescence was fairly established, a small but painful abscess formed in the perineum."

We shall copy the section *Diagnosis* entire. It will be seen that Dr. G. labors to establish a specific difference between typhus and typhoid fevers.

"We now approach an important and, in some respects, the most intricate part of our subject. The questions to be solved are, 1st. Whether there exists any essential diagnostic character between the typhus fever we are describing, and the typhoid fever of Paris, which is also not unfrequent in America. 2nd. If the diagnosis between typhus and certain autumnal remittent fevers attended with extreme prostration, and other typhoid symptoms, be evident. Under the term typhoid we include various nervous symptoms detailed in the preceding pages, including the prostration and dulness of intellect. We of course use the term at present in its popular signification; and although it does not possess the greatest precision, still it is consecrated by long usage, and it would be almost impossible to substitute another epithet equally expressive.

By diagnosis we mean the *comparison* of all the symptoms appreciable by us in disease. This comparison requires a careful examination of the symptoms pretented duiing life, and of the phenomena observed after death, in such cases as terminate unfavorably. We do not base our classification of diseases solely upon their anatomical lesions, although these lesions are oftentimes more constant than any other single symptom whatever; but we group together lesions and symptoms whenever they occur together with sufficient frequency to admit this process of generalization.

In accordance with this signification of diagnosis, we admit the existence of phthisis previously to the formation of tuberculous matter in the same manner as we conceive the possibility of typhoid fever occurring without the characteristic lesion which gives to it the name dothinenteritis.

It is not only necessary to compare the symptoms and the pathological phenomena, but also to examine the succession of the symptoms and their relative intensity at different periods of the disease. It is necessary to attend to this order of symptoms even in those diseases in which, from the development of the local signs, the diagnosis is easy, but in fevers about which so much confusion has existed, as in typhus fever, the most careful observation of all the points is absolutely essential. When we attempt to disentangle this confusion we must group our symptoms, and enquire if one series occurs in a sufficient number of cases for us to admit the succession of the phenomena as nearly, if not quite, constant. If this series does occur in the large majority of cases, the symptoms offer a relation which is sufficiently constant for us to admit them as constituting a distinct disease. If the most important symptoms be present, the absence of one or two, or even a large number of them, does not materially impair the diagnosis; nor does the accidental presence of some irregular symptom which is not usually met with, and does not form a link of the chain, render it uncertain.

We are the more desirous of insisting upon this distinction, as the remarks we made relative to this subject have evidently been misunderstood. The writer to whom we allude supposes that we limit the distinctive characters of the continued fevers purely to their anatomical lesions. We certainly desire to take a more extensive ground; and, from the examination of a large number of facts, we are fully convinced that a set of symptoms which characterize typhoid fever or dothinenteritis, are connected with a lesion of the glands of Peyer. We have met with no exception; and the cases in which the symptoms are said to have been present without the lesion, are confessedly so few in number as to render them doubtful. At most, they prove only that the fever may pass in some rare instances through its course without its anatomical signs being developed. In the same manner turberculous fever occasionally proves fatal before the deposit of the morbid substance from which its name is derived. Now this same lesion of the intestines is certainly so uncommon in other fevers, that at Philadelphia, out of eighteen or twenty cases of malignant remittent, and at least fifty of typhus, I have seen but one example in which the glands of the small intestine were at all affected, although they were always examined with scrupulous care. Even in that solitary instance the lesion was slight, confined to the mucous coat, and did not extend to the cellular substance, as in dothinenteritis.

On considering the symptoms of typhus and typhoid fevers, we observe that the latter disease is not confined to any particular season; it commonly attacks individuals of a particular age, and exposed to some unaccustomed mode of life. It sometimes occurs at the same time that an epidemic of autumnal remittent or of typhus exists. I have seen it under both these circumstances, but I have always observed symptoms which distinguished it from either. There could be no doubt of the correctness of the diagnosis, for it was not made in private practice, but in hospitals, where there were always a number of physicians and pupils present to correct and verify the facts.

These remarks are designed to show that the distinctive characters of these fevers are not such as in practice to allow them to be confounded together. Nor was it very difficult to acquire this facility of diagnosis, as all the better instructed students easily attained it. That the very early stages of typhus and typhoid fevers resemble each other is true, but in no greater degree than in the early stages of typhoid fever and small-pox, which have known to be mistaken for each other by the most experienced observers. When the initial period of the fever is passed, the disease may be readily distinguished. Even very early, before the fever assumes its characteristic appearance; there is usually some fact which may throw light upon its nature.

1. Dothinenteritis is usually a sporadic disease, although it sometimes appears as a wide-spread epidemic. In the latter case the symptoms are so well marked, that these are never doubtful, except in a few of the earliest examples. Now, typhus is very rarely sporadic, and if scattering cases do occur, they are generally connected with an epidemic and follow it, as scattering cases of cholera were observed for a long time after the great epidemic of 1832.

2. Typhus is evidently very contagious; in the epidemic of 1836 it was quite as contagious as small-pox. I am fully convinced of its contagious nature from extensive observation as a physician to the hospital, and from the official visits and inquiries which I made as a member of the Board of Health. Dothinenteritis is certainly not contagious under ordinary circumstances, although in some epidemics we have strong reason to believe that it becomes so. It bears in this respect the same relation to typhus fever that measles does to small-pox.

3. The initial symptoms of the two affections chiefly differ in the greater stupor, dulness and prostration of typhus, which are in strong contrast to the moderate cephalalgia and disturbance of the senses in dothinenteritis.

Still there are now and then, perhaps once in twenty or thirty cases, some symptoms which are apparently common to the two forms of fever. Just as in the diagnosis of measles and scarlatina there is usually no difficulty, but we sometimes see cases of a hybrid character in which the most experienced physicians may be doubtful. In two or three cases out of three hundred, the symptoms of typhus and typhoid fever seemed blended together; but these were slight forms of disease, which are necessarily less distinct than those of a more severe type. In practice, such cases are too rare to give rise to any difficulty.

The more severe cases of dothinenteritis sometime resemble typhus fever very closely, but the resemblance is confined to the symptoms offered by the patient in the most aggravated period of the disease, and does not extend to the succession of symptoms. Indeed, if these cases of typhoid fever are examined at the early stages of the disease, they are certainly more characteristic than the slighter varieties; and although the symptoms occurring during a single day would lead us into error, the comparison of the successive changes will always guide us.

When the disease is completely formed, the characters on which the distinction between the two forms of fevers rest, are—1. The suffusion of the eyes, which occurs in every case, or nearly every case of typhus fever, with the dusky-red aspect of the countenance. 2. The extreme stupor and inactivity of the mind even when positive delirium does not exist. 3. We also observe in typhus no constant abdominal symptom, and at first merely dulness on percussion and feebleness of respiration at the posterior surface of the lungs. 4. If to these symptoms be added the peculiar eruption of petechiæ, which is scarcely ever absent in whites, there remains hardly a possibility of error. In the typhoid fever we consider as distinctive characters, the prostration, the somnolence, the slow development of nervous symptoms, which are not so strongly marked as in typhus. The abdominal symptoms are tympanitis, pains in the abdomen, and diarrhœa. The sibilant rhonchus is heard in the chest; and lastly, there is an eruption of rose-colored papulæ and sudamina upon the skin.

It is not necessary to insist upon the diagnosis between typhus and the ordinary autumnal remittents. The peculiar season at which these latter diseases originate, their progress and termination, all differ too widely from the symptoms of typhus to allow of error, without extreme inaccuracy of observation.

Some rare cases of pneumonia, especially when they occur in drunkards or patients whose constitutions are enfeebled from other causes, resemble typhus in many particulars. Indeed the diagnosis is vastly difficult, were it not for the petechial eruption, as the stupor is sometimes considerable, and the suffusion of the face and eyes nearly as great as in typhus. If in these cases we are totally without knowledge of the early circumstances, we may occasionally mistake a case of pneumonia for typhus fever. But we could scarcely confound the pneumonia, which appears as a mere complication in typyus, with the original inflammation of the lungs. In some of these cases we derive less benefit than we could anticipate from the physical signs, because pneumonia may be present and be readily distinguished by ausculation, but at the same time be strictly secondary. Neither bronchitis nor angina resemble typhus, unless they occur as an epidemic.

We have not room to make an abridgment of the detailed account of the treatment of this epidemic, in which indeed we do not observe any thing particularly new, and shall substitute the author's summary.

The treatment which was usually pursued by us, may be learned from a study of the remedies already indicated; but as their separate examination tends to break up the connexion of this description, we will state in a few words what treatment we thought preferable under ordinary circumstances. At the beginning local blood-letting will diminish the cephalalgia or other local uneasiness which may chance to exist; general bleeding is to be used only as an occasional treatment: afterwards the patient should be kept upon a mild farinaceous diet, with a little animal broth. The heat of the surface is to be moderated by cool or tepid sponging, preferring a solution of the chloride of soda to simple water. The effervescing draught and other mild beverages may be taken as a common drink, more stimulating diaphoretics if the strength of the patient should fail; wine and other stimulants should be given when the prostration is great; and quinine, with a concentrated diet, should be added when the fever subsides, and the skin becomes cool. Emetics, purgatives and blisters were found useful occasional prescriptions, adapted to the removal of particular states of the system, but did not answer our expectations as a general method of treatment.

The general conclusions with the respect to the power of treatment, are, that though it cannot cut short the petechial typhus after the disease is formed, it may shorten the duration, diminish the mortality, and mitigate the severity of the symptoms.

